# Persistence of newly prescribed 5-aminosalicylic acid in patients with ulcerative colitis: A nationwide comprehensive database study

**DOI:** 10.1371/journal.pone.0316181

**Published:** 2024-12-30

**Authors:** Tatsuya Noda, Kotaro Kuwaki, Munehito Machida, Yasuyuki Okumura, Yuichi Nishioka, Tomoya Myojin, Tomoaki Imamura

**Affiliations:** 1 Department of Public Health, Health Management and Policy, Nara Medical University, Nara, Japan; 2 Department of Public Health, Kurume University School of Medicine, Fukuoka, Japan; 3 Division of Gastroenterology, Department of Medicine, Kurume University School of Medicine, Fukuoka, Japan; 4 Department of Public Health Policy, National Institute of Public Health, Saitama, Japan; 5 Initiative for Clinical Epidemiological Research, Tokyo, Japan; National Trauma Research Institute, AUSTRALIA

## Abstract

The 5-aminosalicylic acid (5-ASA) agents are first-line drugs for ulcerative colitis (UC). However, intolerance as well as other issues have been reported for these drugs, making it difficult to sustain this treatment; accordingly, the persistence of 5-ASA is an important indicator of UC treatment strategy. We aimed to analyze the persistence of 5-ASA in patients with UC in Japan. This was a 1-year, nationwide, population-based cohort study using the National Database of Health Insurance Claims and Specific Health Checkups of Japan. We identified patients who were assigned UC-related disease codes and newly prescribed 5-ASA between April 2015 and September 2019 and specified the number of days until 5-ASA prescriptions were interrupted during a follow-up of up to 365 days. Among the 137 million patients who were covered by the universal health insurance in Japan during the study period, 68,234 eligible patients were identified. The 5-ASA persistence in these patients were 87.2%, 65.6%, and 56.4% after 30, 180, and 365 days, respectively. The 5-ASA persistence by subtype at 365 days was 54.4%, 56.4%, and 57.6% for time-dependent, pH-dependent, and multi-matrix system types, respectively. The 5-ASA persistence rate after 365 days was 65.0% for those under 20 years of age, 51.0% for those 20–39 years old, 57.5% for those 40–64 years old, and 65.5% for those over 64 years of age. This study revealed the 1-year persistence of newly prescribed 5-ASA in patients with UC newly prescribed 5-ASA in Japan, based on a national claims database of more than 100 million individuals.

## Introduction

Ulcerative colitis (UC) is a chronic inflammatory bowel disease associated with repeated remissions and relapses [1]. It is one of the most common refractory gastrointestinal system diseases [2]. Prompt induction of remission and long-term maintenance of remission are important [3–6] and persistent treatment of UC is said to improve patient quality of life [7]. In recent years, biologics and molecular-targeted therapies have become the standard of care for moderately to severely active UC [8, 9]. These therapies show a high remission rate and persistence, but there are concerns about the risk of susceptibility to infection and cancer [10–12]. In contrast, 5-aminosalicylic acid (5-ASA), which is relatively inexpensive and safe, is a basic drug used to treat mild to moderate UC, in addition to steroid immunomodulators [13]. Colorectal inflammation is an independent risk for colorectal cancer in patients with UC, and untreated UC leads to a higher incidence of colorectal cancer [14, 15]. 5-ASA is highly useful in the treatment of UC, as it has been suggested that controlling inflammation with 5-ASA lowers the risk of carcinogenesis [16, 17].

While 5-ASA is a first-line drug for UC treatment, approximately 5–10% of patients experience symptoms of allergy or intolerance such as fever, abdominal pain, and bloody stools early in treatment [7, 13, 18, 19]. Therefore, in UC treatment, two key clinical indicators are “adherence”—how well a person follows medication instructions—and “persistence”—how long a person keeps taking their medication [20–22]. Regarding medication adherence, reports from the United States [23–26], Canada [27] and Japan [5] and review articles [28, 29] show a lower relapse rate of UC in the adherence group than in the non-adherence group. On the other hand, persistence is an overall end result of drug discontinuation due to various reasons, and is an important clinical indicator referred to in UC treatment policy considerations and in explaining prescribing policies to patients; however, only a few studies have addressed this issue [27, 30]. Currently, there are three types of 5-ASA (time-dependent, pH-dependent, and multi-matrix system [MMX]), and the prescribed subtype may be switched during treatment. However, all studies on 5-ASA persistence have tracked the persistence of each 5-ASA subtype [27, 30].

In addition, previous studies were limited by regional and patient population bias, sample size, and the analysis of a large population. Accordingly, an unbiased sample population, such as a large-scale claims database, is desirable. Japan, with a population of 127 million, achieved universal health coverage in 1961, and has a health system in which all citizens are covered under some form of medical insurance [31]. The claims information for these medical insurance plans is integrated into a national database called the National Database of Health Insurance Claims and Specific Health Checkups of Japan (NDB). Because the same claim forms are used by all medical institutions by any kind of insurer for reimbursement in Japan [32], the NDB is one of the largest medical visit databases in the world, storing data from visits by all national citizens, approximating 110 million visits per year. The coverage of the entire Japanese population and accessibility of medical care in Japan result in minimal biases and omissions, which greatly benefit the descriptive epidemiology of disease treatment.

Although the prevalence of UC in Japan is lower than that in European and North American countries [33], the number of patients with UC in Japan and other Asian countries is on the rise [34–36]. Inflammatory bowel disease is a global disease with accelerated morbidity in societies undergoing industrialization and modernization [37]. Therefore, a large-scale analysis of 5-ASA medication persistence in Japan would serve as an international reference as countries have rapidly growing populations.

There have been several studies on 5-ASA persistence using receipt databases [27, 30]; however, most studies are limited to specific hospital groups or regions. In addition, there are few studies that are all-inclusive and less influenced by patients’ visit backgrounds [30]. The absence of observational studies utilizing national-scale data from a population of 100 million individuals reduces the chances of overlooking potential patients.

To ascertain how long 5-ASA is used in actual clinical practice, this study aimed to track the overall persistence of 5-ASA prescriptions in addition to the persistence of each 5-ASA subtype in nearly all Japanese patients with UC using the NDB, one of the largest claims databases in the world.

## Methods

**Fig 1:**
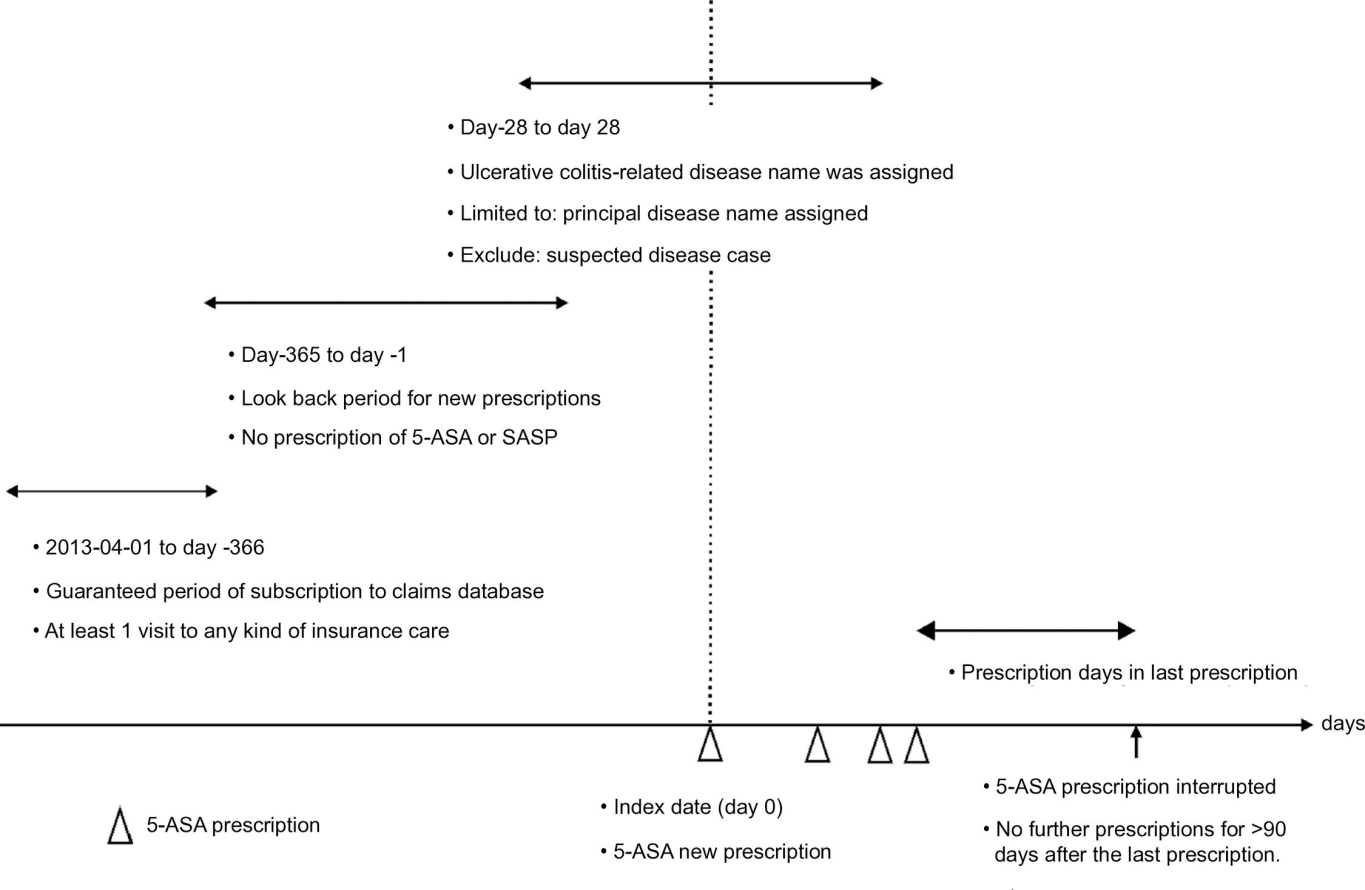
Design diagram: Definition of the inclusion and exclusion of monitored patients and the termination of prescription. 5-ASA, 5-aminosalicylic acid.

This observational study evaluated data from the NDB, the national claims database in Japan, from April 2015 to September 2019. This study meets the criteria of a retrospective study of claims database. The data were accessed for research purposes on 2021-03-14 and authors did not have access to information that could identify individual participants during or after data collection. The sample sizes of studies using Japanese claims data vary widely. The data used in the present study cover over 100 million people which, to the best of our knowledge, is the largest sample not only in Japan but also in the world. In addition, we adopted a novel approach by utilizing technology that allows the tracking of patient visits to multiple medical institutions for the treatment of the same disease [38], and we believe that we can accurately track almost all patients with UC in Japan. Therefore, this study captures trends in UC consultations with a large sample and very small level of selection bias. Patients were identified as patients with UC newly prescribed 5-ASA if they met all the following criteria using standard methodology for database-based persistence estimates (Fig 1):

1. At least one oral 5-ASA preparation was prescribed during the observation period; the first prescription date of the 5-ASA preparation was defined as the index date (day 0).
2. Between day -28 and day 28, UC-related disease was assigned as the primary injury or disease at the medical institution that prescribed the 5-ASA preparation. If the disease name was assigned as a “suspected disease” (i.e., a tentative disease name) by a physician, the patient was excluded. The principal disease name was the disease on which the physician placed the greatest emphasis in the treatment of the patient and was included as the “principal wound or disease” in regular-type medical claims. In Diagnosis Procedure Combination (DPC)-type claims [39], we recognized the principal name filled in the entry blank, “medical resource disease 1,” “medical resource disease 2,” “principal wound or disease,” or “disease name at the time of admission,” which indicates the disease for which the greatest amount of medical resources was invested.
3. Between day -365 and day -1, no oral or topical 5-ASA, salazosulfapyridine (SASP), has been prescribed at any time (guaranteed as “newly prescribed”).
4. Patients with a record of at least one visit to any kind of insurance care between the start date of our NDB data (April 1, 2013) and day -366. The type of disease was not limited to UC.

The 90-day grace period (the maximum interval between prescriptions at which a drug can be continuously administered [40]) was set to allow for the possibility of a temporary interruption of visits due to non-medical events. A grace period was introduced to allow for some degree of noncompliance and irregular dispensing owing to stockpiling. 5-ASA treatment persistence was monitored for 365 days after the index date. For patient tracking, we applied a method that combined several tracking variables originally included in the NDB to improve the tracking rate [38]. For death case ascertainment, we used a method that combined information on deaths in the claims data with information on medical procedures performed immediately before death to improve the identification rate of the NDB [41]. The codes in the NDB we used are given in the supporting information (S1 File).

In addition to describing the basic attributes of patients who received 5-ASA prescriptions and the number of patients by fiscal year (April to the next March), we determined annual changes in the percentage of patients prescribed 5-ASA by subtype. As the main outcome of this study, the persistence rate of 5-ASA was calculated for 5-ASA overall, by subtype, and by age group.

JMP (ver. 15.2.1; SAS Institute, Cary, NC, USA) was used for statistical analysis. Permission to conduct this study was provided by the Nara Medical University Ethics Committee (approval no. 2831) on October 30, 2020. The requirement for informed consent was waived due to the law in Japan. Data were obtained on 2021-03-14 and anonymized before being analyzed.

## Results

**Fig 2:**
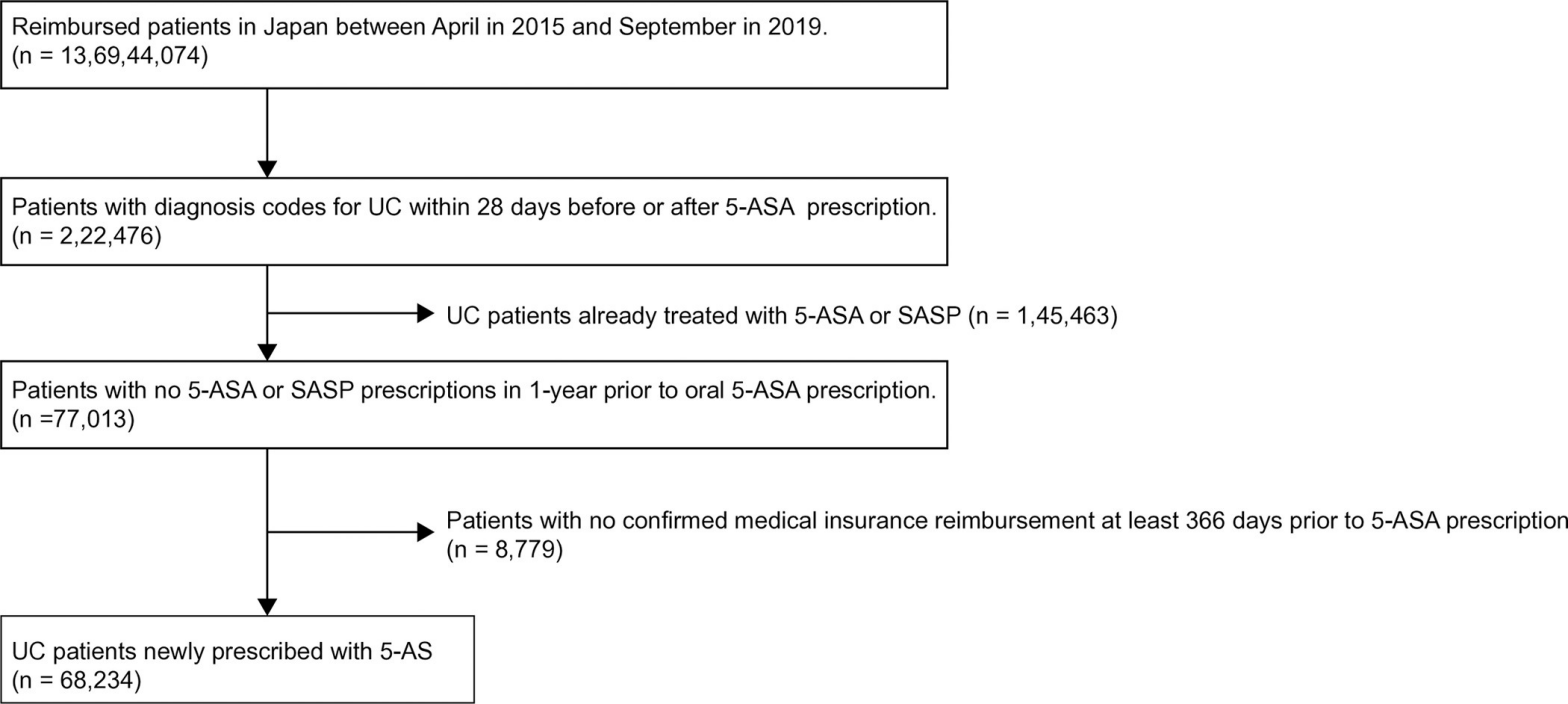
Flowchart of patient selection. 5-ASA, 5-aminosalicylic acid; SASP, salazosulfapyridine; UC, ulcerative colitis.

A flowchart of the extraction method used in this study is shown in Fig 2. We identified 220,000 patients with a UC diagnosis within 28 days before or after a 5-ASA prescription from 137 million citizens who received medical services under the universal health insurance system in Japan. The study period was from April 2015 to September 2019, covering 4 years and 6 months. The study identified 68,234 patients as new users of 5-ASA after excluding those with a history of prior 5-ASA/SASP prescriptions and those whose enrollment in the database could not be verified.

**Fig 3:**
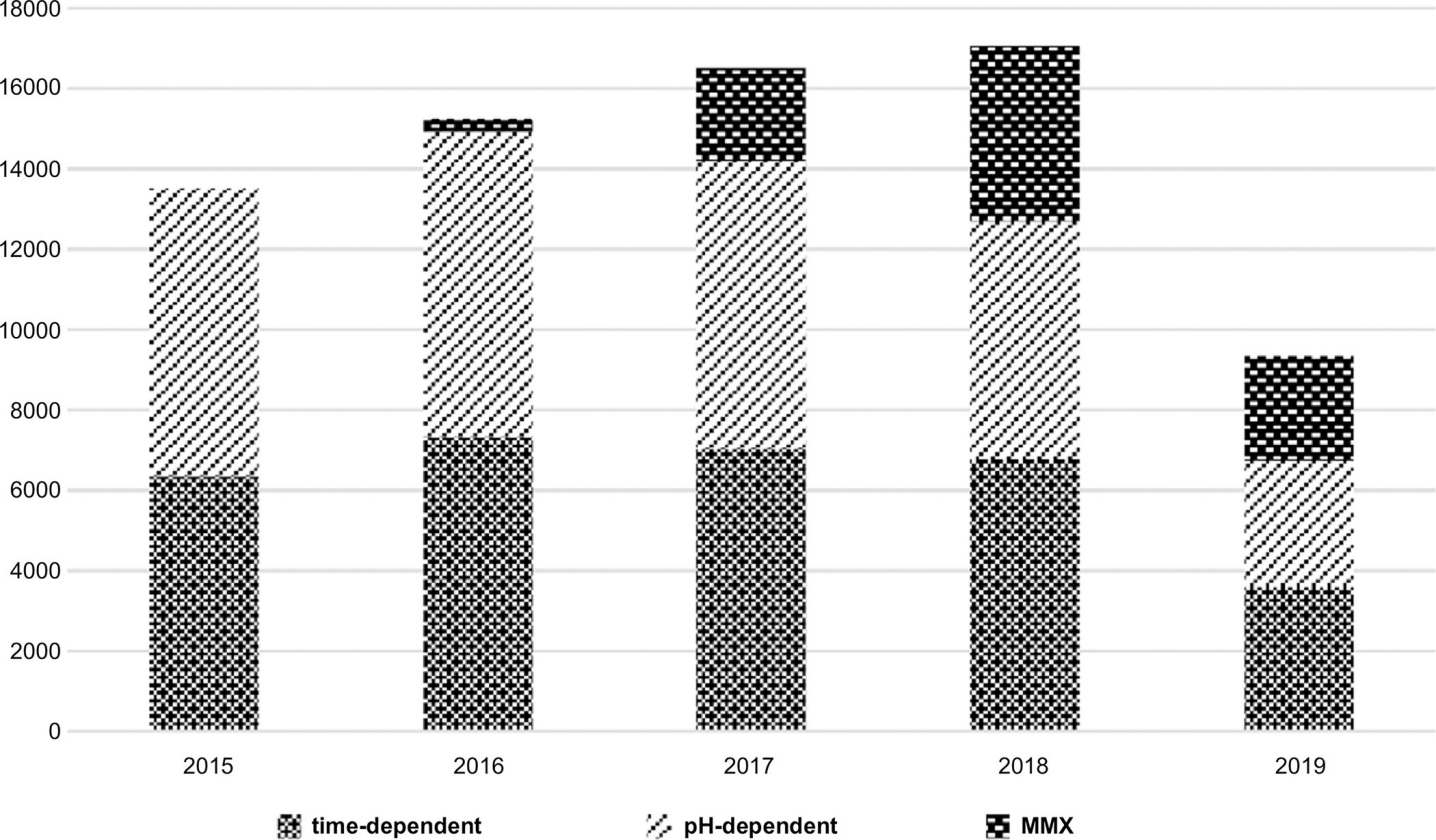
Distribution of starting year by 5-ASA (5-aminosalicylic acid) subtype. *Data correspond to a 6-month period (April 2019 to September 2019). MMX, multi-matrix system.

The basic attributes of the subjects (Table 1) were an average age of 43.3 years, a proportion of men of 56.5%, and a number of patients receiving new prescriptions that was approximately 0.1% of the Japanese population, which has been increasing annually. The breakdown of 5-ASA first prescribed by subtype (Fig 3) showed that the pH-dependent type accounted for 53.2% in fiscal year (FY) 2015, more than the time-dependent type (46.8%); however, the MMX has been rapidly increasing since FY 2016.

**Table 1:** Attributes by age, sex, and fiscal year of the patients monitored in this study.

Overall	68234
**Mean age (SD)**	43.2 (16.7)
**Male %**	56.5
**FY 2015**	13493
**FY 2016**	15216
**FY 2017**	16500
**FY 2018**	17031
**FY 2019 (6 mo.)**	9304

Data are presented as the number of people, unless otherwise mentioned

SD, standard deviation; FY, fiscal year

The persistence rates for newly prescribed 5-ASA in patients with UC are shown in Table 2. Persistence rates for 5-ASA overall after 30, 180, and 365 days were 87.2%, 65.6%, and 56.4%, respectively. The persistence rate for 5-ASA after 365 days by subtype was 54.4% for time-dependent, 56.4% for pH-dependent, and 57.6% for MMX.

**Table 2:** Persistence (%) for the subtype and selected days from the start of initial administration of 5-ASA.

5-ASA subtype	Days after first 5-ASA prescriptions					
	0	30	90	180	365	500
**ALL**	68234	59481	50532	44769	38481	37325
	100%	87.2%	74.1%	65.6%	56.4%	54.7%
		(86.9–87.4)	(73.7–74.4)	(65.3–66.0)	(56.0–56.8)	(54.3–55.1)
**Time**	29783	25584	21438	18870	16197	15676
	100%	85.9%	72%	63.4%	54.4%	52.6%
		(85.5–86.3)	(71.5–72.5)	(62.8–63.9)	(53.8–54.9)	(52.1–53.2)
**pH**	29792	26106	22081	19542	16814	16342
	100%	87.6%	74.1%	65.6%	56.4%	54.9%
		(78.2–88.0)	(73.6–74.6)	(65.1–66.1)	(55.9–57.0)	(54.3–55.4)
**MMX**	9557	8378	7295	6482	5506	5335
	100%	87.7%	76.3%	67.8%	57.6%	55.8%
		(87.0–88.3)	(75.5–77.2)	(66.9–68.8)	(56.6–58.6)	(54.8–56.8)

5-ASA, 5-aminosalicylic acid; MMX, multi-matrix system

Numbers represent patients with persistence of 5-ASA prescriptions at that time.

Percentages indicate the persistence rate.

Numbers in parentheses are 95% confidence intervals for persistence rates.

**Fig 4:**
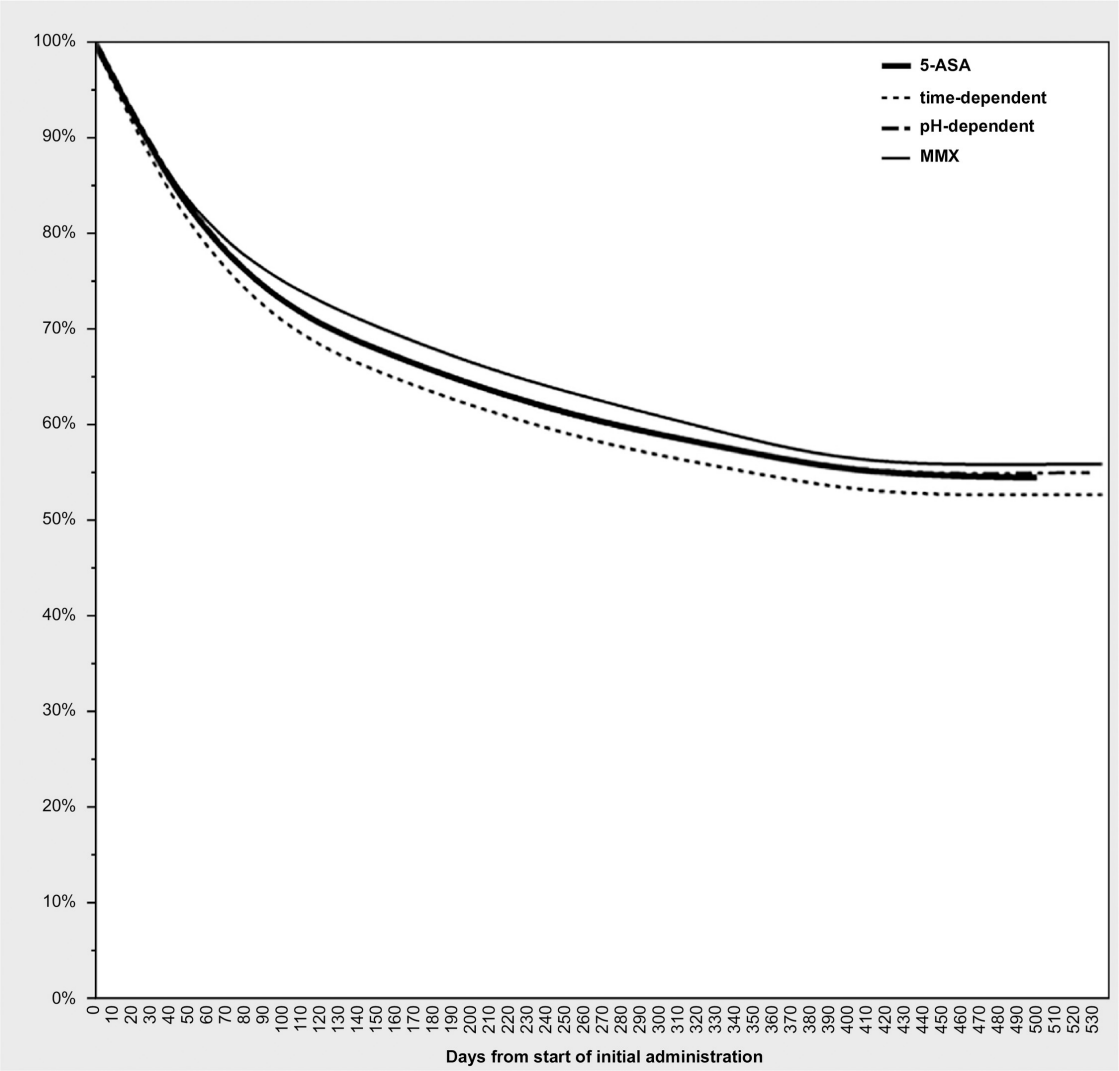
Persistence of 5-ASA (5-aminosalicylic acid). 5-ASA, 5-aminosalicylic acid; MMX, multi-matrix system.

Table 3 shows the 5-ASA persistence rates by age group. Persistence rates after 365 days were higher for those under 20 (65.0%) and over 64 (65.5%) than for those 20–39 (51.0%) and 40–64 (57.5%) years of age (Fig 4). Details on days after 5-ASA prescription and the number of patients persisting with 5-ASA are shown in supporting information file (S1 Table).

**Table 3:** Persistence (%) by age group and selected days from the start of initial administration of 5-ASA.

age	Days after first 5-ASA prescriptions					
	0	30	90	180	365	500
**< 20**	4389	3876	3483	3232	2851	2781
	100%	88.3%	79.4%	73.6%	65.0%	63.4%
		(87.3–89.2)	(78.1–80.5)	(72.3–74.9)	(63.5–66.4)	(61.9–64.8)
**20–39**	27293	23498	19482	16853	13909	13397
	100%	86.1%	71.4%	61.7%	51.0%	49.1%
		(85.7–86.5)	(70.8–71.9)	(61.2–62.3)	(50.4–51.6)	(48.5–49.7)
**40−64**	27900	24438	20783	18424	16052	15596
	100%	87.6%	74.5%	66.0%	57.5%	55.9%
		(87.2–88.0)	(74.0–75.0)	(65.5–66.6)	(57.0–58.1)	(55.3–56.5)
**> 64**	8652	7669	6784	6260	5669	5551
	100%	88.6%	78.4%	72.4%	65.5%	64.2%
		(88.0–89.3)	(77.5–79.3)	(71.4–73.3)	(64.5–66.5)	(63.1–65.2)

Numbers represent patients with persistence of 5-ASA prescriptions at that time.

Percentages indicate the persistence rate.

Numbers in parentheses are 95% confidence intervals for persistence rates.

## Discussion

To the best of our knowledge, this is the first study to use a nationally representative medical database to calculate the number of newly treated patients with UC and the persistence of treatment with 5-ASA. The strengths of this study are as follows: 1) Japan has “universal health insurance,” and the NDB is one of the largest claims databases in the world, covering a population of 100 million, the entire Japanese population; 2) this study tracked the treatment of UC in healthcare facilities throughout the country, including facilities outside specialized gastroenterology hospitals. While previous Japanese studies [5, 42] could not eliminate the effects of recall and selection biases due to questionnaire and medical record surveys conducted at a limited number of facilities, the present study was less affected by these biases.

This study showed a 55.5% persistence after 1 year in patients newly prescribed 5-ASA, which is a higher persistence rate than that reported in previous studies [8, 18]. This difference is because the previous studies evaluated the persistence of one type of medicine for UC treatment, while this study evaluated the total 5-ASA persistence for each patient.

The trends over time in prescription rates by 5-ASA subtype show decreases in pH-dependent 5-ASA, a flat pattern in time-dependent 5-ASA, and an increase in MMX. This could be due to lower dosing frequency and higher maximum doses for time-dependent 5-ASAs than for pH-dependent 5-ASAs in Japan as well as to the growing preference for fewer doses of MMX. The decline in 5-ASA persistence to 12.8% in the first 30 days suggests discontinuation of prescriptions caused by intolerance or allergy to 5-ASA or by a change in diagnosis. The results of this study are consistent with prior research showing that intolerance or allergy to 5-ASA occurs in 5–10% of patients in the first month of treatment [18]. Regarding persistence rates by 5-ASA subtype, no major differences were observed, and it was assumed that adequate treatment was provided for symptoms and patient needs. This is the first large-scale study to observe 5-ASA persistence rates by age group, and the rates were relatively high among younger and older patients. A previous study found that adherence, an indicator similar to persistence rates, was lower for those younger than 40 years of age, and the results of this study do not contradict this [43] In our study, the persistence rate was high for those under 20 years old, which could be attributed to parental involvement in hospital visits and medication management as well as to the active policies in Japan to subsidize medical costs for children.

The fact that the majority of patients continue to use an inexpensive and effective drug, such as 5-ASA, as a treatment for UC after 1 year emphasizes the need to use 5-ASA as a first-line treatment; this will contribute to numerical persistence based on large-scale data for patients being newly initiated on treatment [44]. However, the results of this study indicate an upper limit for the rate of prescription interruptions due to 5-ASA intolerance. The “incidence of 5-ASA intolerance leading to prescription interruptions” within the first year of treatment should not exceed 44.5%.

In Japan, MMX-type 5-ASA was included in the universal insurance coverage in November 2016 and has since been used in patients with UC. The number of patients by formulation and year showed that prescriptions of MMX-type 5-ASA have increased since 2016, which may be because they have the highest maximum daily dose (4,800 mg) compared to that of the other two types. They are administered once a day, which is expected to improve persistence.

This study had certain limitations. The first is the validity of 90 days as the period during which 5-ASA prescriptions were considered interrupted. In the sensitivity analysis, we observed an impact on the discontinuation rate when the period was set to 30 and 60 days, and the persistence rates after 365 days were 43.5% and 53.4%, respectively. Accordingly, adequate periods of 60 and 90 days were considered. Second, our data source did not include medical care that was not covered by the statutory health insurance system. The largest group outside the system comprises social welfare recipients, which represent 1.63% of the Japanese population [45], which is unlikely to significantly distort the present results. Third, this study could not rule out the possibility that the prescribed 5-ASA was not administered internally. However, from the standpoint of drug payment costs, it is likely that few patients would be prescribed 5-ASA for a long period of time but would not consume it. Fourth, we could not identify the reasons for the discontinuation of 5-ASA prescriptions. Possible reasons may include the occurrence of intolerance, remission of symptoms, or the patient’s own decision. The purpose of this study was to provide a clinical prescription for persistence that encompasses all these reasons. It should be noted that the discontinuation of visits for economic reasons is less common in Japan than in other countries [46], and the proportion of discontinuation for biological reasons for prescription is probably higher. Fifth, diagnosis was defined in this study by the disease name plus prescription drug, which may have not fully reflected changes in disease severity at the time of diagnosis or during the subsequent clinical course. In this regard, since more than 90% of UC cases in Japan are mild to moderate and 5-ASA is the first-line drug in many cases, even in severe cases, the impact of using the start of the prescription as the definition of disease is not considered significant.

This study is the first to reveal the number of patients who were newly treated with 5-ASA for UC, treatment persistence, and subsequent treatment transitions using a nationally representative database covering the majority of the Japanese population. The 5-ASA persistence rate for 1 year was 55.5%, which is higher than that reported in previous studies. There was little difference in persistence by formulation, suggesting that prescriptions have been tailored to patient backgrounds in Japan in recent years.

## Conclusion

This study provides an important indicator that will contribute to the development of future treatment strategies for UC using 5-ASA. Research on persistence is lacking; therefore, studies that consider racial and regional differences are desirable. Further research on the follow-up of patients who discontinued treatment is required.

## Supporting information

S1 File NDB codes used in this study. (DOCX)

S1 Table Persistence for the subtype and selected days from the start of initial administration of 5-ASA. (PDF)
