# Supplementary material for: Persistence of newly prescribed 5-aminosalicylic acid in patients with ulcerative colitis: A nationwide comprehensive database study
Source: PLoS One. 2024 Dec 30;19(12):e0316181. doi: 10.1371/journal.pone.0316181 (PMC11684613; doi:10.1371/journal.pone.0316181)
Supplement: S1 File — (DOCX) [file pone.0316181.s001.docx]

**Supplementary Information**

NDB codes used in this research

**Persistence of newly prescribed 5-aminosalicylic acid in patients with ulcerative colitis: A nationwide comprehensive database study**

Tatsuya Noda, MD, PhD^1,#,*^, Kotaro Kuwaki, MD, PhD ^2,3,#^, Munehito Machida, MD, PhD ^4,#^, Yasuyuki Okumura, PhD ^5^, Yuichi Nishioka, MD, PhD ^1^, Tomoya Myojin, MD, PhD ^1^, Tomoaki Imamura, MD, PhD ^1^

^1^Department of Public Health, Health Management and Policy, Nara Medical University, Nara, Japan

^2^Department of Public Health, Kurume University School of Medicine, Fukuoka, Japan

^3^Division of Gastroenterology, Department of Medicine, Kurume University School of Medicine Fukuoka, Japan

^4^Department of Public Health Policy, National Institute of Public Health, Saitama, Japan

^5^Initiative for Clinical Epidemiological Research, Tokyo, Japan

^*^**Correspondence:** Tatsuya Noda, MD, PhD

Email: [noda@naramed-u.ac.jp](mailto:noda@naramed-u.ac.jp)

To ensure the reproducibility of the findings of this research, the NDB codes we used (as of December 2022) are shown below. NDB codes are defined by the Ministry of Health, Labour, and Welfare, Japan. (Available only in Japanese.)

The data underlying this article are made available in accordance with guidelines for health care database research issued by the joint ISPE-ISPOR Special Task Force on Real World Evidence in Health Care Decision Making, at <https://doi.org/10.1016/j.jval.2017.08.3018> .

- Ulcerative Colitis-related disease name code

5569003,8845754,8845755,8845756,8845757,8845758,8845759,8845760,8850127,8831064,8845766,8845767,0091015,8845772,8845779,8845782,8845785,8845793,8845798,8844992,8845810,8845816,8845833,5551002,5551005,5694035

- Drug code

oral 5-ASA (time-dependent types)

620008106,620008107,620008510,620008511,620009440,620009441,620506801,621867201,621884302,621884303,621890601,621965201,622011801,622026201,622053401,622065002,622102902,622102903,622109402,622143201,622143301,622165801,622173401,622442701,620008108,620008509,620008512,620008513,620008514,622102901,622109401,622129401,622129501

oral 5-ASA (pH-dependent types)

621969902,622416401,622458101,622537801,622563401,622590701

oral 5-ASA (multi-matrix system type; MMX)

622517001

SASP

620008624,620009119,621133002,622023001,622746800,610453008,610453051,610453065,610463004,620004897,620006949,621350101,621442802,621442905,621443001,621443002,622039001,622039002,622103601,622103602,622161401,622161402,622173801,622173901,622746900,622747000,622833801

topical formulation of 5-ASA or SASP

622167101,622234801,622818901,660470002,620008997
