# Supplementary material for: Persistence of newly prescribed 5-aminosalicylic acid in patients with ulcerative colitis: A nationwide comprehensive database study
Source: PLoS One. 2024 Dec 30;19(12):e0316181. doi: 10.1371/journal.pone.0316181 (PMC11684613; doi:10.1371/journal.pone.0316181)
Supplement: S1 Table — (PDF) [file pone.0316181.s002.pdf]

S2 Supplementary Table

Persistence for the subtype and selected days from the start of initial administration of 5-ASA

Persistence of newly prescribed 5-aminosalicylic acid in patients with ulcerative colitis: A nationwide comprehensive database study

Tatsuya Noda, MD, PhD[1,#,*], Kotaro Kuwaki, MD, PhD [2,3,#], Munehito Machida, MD, PhD [4,#], Yasuyuki Okumura, PhD [5], Yuichi Nishioka, MD, PhD [1], Tomoya Myojin, MD, PhD [1], Tomoaki Imamura, MD, PhD [1]

[*]**Correspondence:** Tatsuya Noda, MD, PhD

Email: noda@naramed-u.ac.jp

Original data in Table 2 and Figure 4.

- Legend for the table below

Patient numbers in the blanks are not to be disclosed according to legal regulations.
Each subtype can be prescribed to the same patient at the same time.

5-ASA: 5-aminosalicylic acid
Time: Time-dependent type 5-ASA
pH: pH-dependent type 5-ASA
MMX, multi-matrix system

Number of patients persisting 5-ASA according to days after prescription

Days after prescription	5-ASA_ALL	Time	pH	MMX
initial	68234	29783	29792	9557
1	68142	29736	29757	
2	68099	29719	29739	9533
3	68039	29683	29722	
4	67936	29613	29693	9519
5	67893	29589	29679	
6	67304	29256	29464	9461
7	67240	29222	29438	
8	67195	29196	29422	
9	66987	29074	29344	9439
10	66908	29029	29319	9429
11	66831	28987	29291	
12	66750	28946	29260	9411
13	64689	28008	28380	9090
14	64538	27931	28323	9069
15	64441	27887	28287	9050
16	64349	27840	28260	9026
17	64244	27782	28231	9000
18	64130	27726	28187	8984
19	63920	27616	28099	8962
20	63400	27382	27879	8880
21	63272	27329	27825	8856
22	63157	27270	27787	8836
23	63052	27220	27741	8818
24	62927	27171	27685	8790
25	62799	27111	27638	8765
26	62678	27057	27586	8749
27	61195	26397	26908	8538
28	61049	26336	26853	8504
29	59598	25635	26157	8394
30	59481	25584	26106	8378
31	59383	25537	26072	8360
32	59288	25501	26032	8339
33	59187	25460	25984	8323
34	58775	25281	25797	8259
35	58649	25217	25742	8247
36	58539	25164	25699	8231
37	58434	25123	25650	8213
38	58325	25068	25605	8196
39	58189	25003	25542	8184
40	58065	24945	25486	8171
41	57614	24743	25276	8111
42	57500	24697	25220	8096
43	57338	24632	25137	8076
44	57217	24568	25093	8060
45	57109	24521	25045	8046

46	57021	24483	25003	8035
47	56923	24431	24963	8025
48	56709	24339	24862	7995
49	56579	24276	24810	7975
50	56478	24234	24761	7961
51	56401	24206	24721	7947
52	56332	24174	24691	
53	56261	24132	24673	7926
54	56153	24089	24627	7906
55	55458	23752	24320	7827
56	55367	23711	24284	7808
57	55223	23656	24214	7788
58	55137	23610	24183	7778
59	54520	23294	23902	7735
60	54449	23262	23872	7723
61	54366	23226	23831	
62	54060	23078	23692	7681
63	53982	23046	23653	7671
64	53886	23009	23603	
65	53819	22984	23567	7654
66	53757	22955	23538	
67	53690	22923	23512	7637
68	53602	22883	23473	
69	53331	22749	23359	7593
70	53247	22710	23321	7583
71	53168	22676	23286	7567
72	53086	22639	23249	7556
73	52986	22598	23200	7544
74	52924	22571	23174	7531
75	52858	22545	23144	7519
76	52666	22453	23065	7495
77	52587	22418	23029	7483
78	52518	22386	22998	
79	52440	22346	22964	7468
80	52368	22312	22939	7454
81	52297	22284	22907	7437
82	52240	22254	22885	
83	51891	22090	22722	7403
84	51813	22049	22689	
85	51731	22016	22647	7386
86	51654	21982	22611	
87	51557	21939	22571	7361
88	51485	21913	22533	
89	50774	21543	22196	7322
90	50532	21438	22081	7295
91	50458	21402	22048	
92	50371	21362	22010	7277
93	50317	21335	21990	
94	50235	21302	21947	7260
95	50190	21283	21928	
96	50125	21253	21905	7238

97	49885	21158	21780	7214
98	49734	21075	21717	7204
99	49653	21031	21695	7188
100	49608	21009	21675	
101	49536	20976	21649	7168
102	49473	20949	21614	
103	49414	20921	21590	7154
104	49289	20858	21543	7130
105	49223	20829	21513	
106	49155	20794	21485	7111
107	49113	20774	21462	
108	49048	20743	21437	7095
109	48999	20723	21416	7085
110	48951	20703	21393	
111	48781	20617	21321	7061
112	48717	20595	21286	
113	48652	20562	21260	7044
114	48593	20535	21237	
115	48528	20507	21208	7026
116	48471	20485	21179	7016
117	48397	20444	21150	
118	48270	20385	21094	6997
119	48195	20350	21060	
120	48132	20324	21031	6979
121	48086	20303	21012	
122	48021	20272	20981	6966
123	47970	20254	20960	6954
124	47909	20227	20937	6941
125	47786	20178	20886	6917
126	47727	20154	20862	6904
127	47685	20139	20840	
128	47637	20120	20822	6887
129	47590	20106	20795	
130	47554	20086	20783	6876
131	47505	20061	20766	
132	47391	20007	20714	6856
133	47340	19980	20693	
134	47293	19962	20672	6844
135	47238	19933	20654	
136	47194	19920	20627	6831
137	47143	19901	20599	
138	47093	19876	20580	6814
139	46946	19806	20522	6788
140	46895	19778	20503	
141	46845	19752	20483	
142	46804	19740	20459	6774
143	46753	19707	20447	
144	46723	19696	20431	6763
145	46676	19672	20407	
146	46578	19637	20357	6744
147	46528	19615	20334	

148	46487	19596	20315	6731
149	46448	19585	20291	
150	46385	19565	20256	6719
151	46335	19541	20231	
152	46296	19520	20215	
153	46187	19475	20166	6698
154	46144	19456	20142	
155	46091	19433	20116	
156	46058	19420	20101	6687
157	46004	19394	20079	
158	45967	19382	20057	6675
159	45918	19360	20036	
160	45837	19332	19991	6658
161	45792	19311	19976	6648
162	45734	19289	19948	
163	45684	19270	19929	6626
164	45637	19249	19906	
165	45598	19232	19891	6613
166	45547	19207	19870	
167	45436	19162	19819	6591
168	45389	19141	19803	6581
169	45353	19126	19786	
170	45313	19111	19768	6568
171	45261	19093	19741	
172	45229	19080	19726	6555
173	45184	19055	19711	
174	45048	18985	19663	6529
175	45007	18967	19644	
176	44954	18948	19623	6511
177	44920	18934	19612	
178	44883	18918	19598	6495
179	44834	18895	19576	
180	44769	18870	19542	6482
181	44665	18832	19486	6470
182	44626	18815	19467	
183	44575	18794	19443	6460
184	44539	18779	19427	
185	44490	18762	19400	6448
186	44453	18746	19380	
187	44408	18732	19357	6436
188	44331	18691	19328	
189	44295	18669	19315	6424
190	44259	18651	19301	
191	44222	18628	19289	
192	44180	18614	19267	6410
193	44143	18595	19251	
194	44100	18577	19233	6400
195	44013	18545	19191	6384
196	43967	18527	19170	
197	43919	18510	19143	
198	43881	18494	19125	6365

199	43839	18472	19111	
200	43812		19100	6352
201	43775	18447	19084	
202	43711	18412	19061	6336
203	43680	18401	19046	
204	43635	18383	19028	6322
205	43613	18373		
206	43577	18358	19003	6311
207	43536	18346	18979	
208	43494	18329	18964	6295
209	43418	18297	18931	6285
210	43383	18280	18918	
211	43346	18265	18902	6273
212	43313	18249	18889	
213	43271	18226	18876	6262
214	43235	18208	18863	
215	43201	18198	18844	6251
216	43127	18167	18811	6237
217	43098	18154	18795	
218	43064	18137	18782	
219	43029	18121	18767	
220	42994	18109	18745	
221	42959	18096	18729	6221
222	42925	18084	18710	
223	42832	18048	18663	6208
224	42798	18032	18653	
225	42758	18014	18633	6196
226	42720	18000	18614	
227	42680	17980	18596	
228	42657		18584	6185
229	42615	17953	18564	
230	42541	17920	18527	6172
231	42512	17906	18514	
232	42479	17893	18502	6160
233	42445	17881	18488	
234	42418	17869		6143
235	42386	17851	18475	
236	42351	17838	18454	
237	42282	17809	18420	6126
238	42248	17790	18408	
239	42202	17765	18390	6115
240	42172	17752	18375	
241	42144	17739	18361	
242	42114	17726	18346	
243	42072	17708	18327	6103
244	41988	17674	18287	6092
245	41955	17663	18267	
246	41931	17653		
247	41909		18247	6079
248	41882	17638	18233	
249	41845	17623	18215	

250	41819	17613	18201	6069
251	41750	17578	18175	
252	41724	17564		6058
253	41696	17552	18153	
254	41668	17539	18140	
255	41632	17523	18125	6046
256	41608	17511	18115	
257	41566	17492	18098	
258	41509	17465	18076	6029
259	41479	17452		
260	41448	17441	18053	6016
261	41422	17430	18040	
262	41382	17411	18024	
263	41356	17400	18011	6005
264	41320	17383	18001	
265	41267	17361	17977	5988
266	41233	17346	17961	
267	41211		17951	5978
268	41180	17324	17938	
269	41147	17312	17923	
270	41128			5967
271	41090	17287	17897	
272	41031	17261	17874	5954
273	40998	17249	17856	
274	40972		17844	
275	40945	17230	17833	5940
276	40918	17212		
277	40894	17200	17819	
278	40856	17187	17803	5922
279	40799	17162	17778	
280	40766	17147	17767	5908
281	40739	17136	17757	
282	40721			5898
283	40698	17115	17741	5898
284	40680			
285	40645	17093	17715	
286	40590	17065	17701	5880
287	40562	17054	17689	
288	40533		17674	5868
289	40506	17032	17663	
290	40477	17018	17650	
291	40464			
292	40430	16994	17633	5856
293	40376	16974	17605	
294	40351	16960	17594	
295	40333		17584	5846
296	40311	16949		
297	40290	16938	17568	5836
298	40270	16926		
299	40242	16913	17553	
300	40198	16891	17539	5820

301	40170	16876		
302	40147		17525	5807
303	40122	16856		
304	40094		17501	5797
305	40076	16837		
306	40052	16826	17485	
307	40009	16813	17463	5784
308	39990		17450	
309	39962	16792		
310	39934	16781	17434	5769
311	39912		17420	
312	39897	16770		
313	39873		17403	5757
314	39831	16742	17389	
315	39809		17379	5743
316	39787	16726	17366	
317	39764	16715		
318	39741		17344	
319	39721	16701		5730
320	39701	16690	17329	
321	39658	16671	17311	
322	39633	16660	17300	5720
323	39605	16649		
324	39586		17280	5710
325	39564	16631		
326	39543		17260	
327	39517	16615	17247	5700
328	39473	16597	17228	
329	39449		17217	5683
330	39420	16583	17205	
331	39399	16573	17195	
332	39372	16563	17181	5670
333	39348	16552		
334	39318	16538	17162	5660
335	39267	16515	17146	5647
336	39247		17135	
337	39224	16495		
338	39212		17125	5637
339	39193	16481		
340	39170	16470	17111	
341	39136	16456	17097	5624
342	39089	16436	17081	5613
343	39070			
344	39051	16416	17066	
345	39029			
346	39007	16396	17052	5599
347	38989	16386		
348	38967	16376	17037	
349	38904	16357	17006	5580
350	38889			
351	38870	16345	16987	

352	38842		16971	
353	38805	16329	16953	5562
354	38784		16943	
355	38755	16307	16932	
356	38705	16284	16909	
357	38686			5541
358	38664	16273	16895	
359	38640		16883	5531
360	38622	16256		
361	38596		16863	
362	38566	16235	16848	5521
363	38518	16213	16827	
364	38500			
365	38481	16197	16814	5506
366	38464			
367	38445	16181	16799	
368	38422	16168		5495
369	38398		16781	
370	38349	16138	16761	
371	38330			5481
372	38307	16114	16750	
373	38285		16740	
374	38264	16100	16730	5469
375	38248			
376	38218	16083	16708	
377	38178	16063	16696	5454
378	38158		16683	5454
379	38133	16041		
380	38112		16669	
381	38090	16022	16659	
382	38075			5440
383	38054	16008	16641	
384	38019	15995	16627	
385	38003	15985		5428
386	37993			
387	37965	15971	16607	
388	37950			5415
389	37926	15952	16593	
390	37907		16583	
391	37864	15930	16563	
392	37850			5400
393	37828	15911	16551	
394	37809	15901		
395	37794		16535	
396	37783	15888		
397	37768			
398	37743	15865	16516	
399				
400	37716	15852	16505	5389
401				
402	37695		16493	

403	37685	15839		
404	37668		16481	
405	37652	15826		
406				5377
407	37639			
408			16470	
409	37618	15812		
410	37608			
411	37595		16455	5367
412	37573	15791	16445	
413	37559			
414			16435	
415	37545			
416	37532	15776		
417	37522		16424	
418	37512	15766		5355
419	37486	15756	16406	
420				
421	37469	15746		
422				
423	37454		16393	
424				
425		15736		
426	37434			
427				
428				
429			16381	
430	37424			
431				
432		15719		
433	37403			5345
434	37403			5345
435				5345
436				5345
437			16368	5345
438	37393			
439		15707		
440	37374			
441				
442				
443			16354	
444	37360	15696		
445		15696		
446				
447				
448				
449	37344			
450	37344			
451				5335
452		15686		5335
453				5335

454				5335
455				5335
456				5335
457				5335
458				5335
459				5335
460				5335
461				5335
462				5335
463				5335
464		15676		5335
465		15676		5335
466		15676		5335
467		15676		5335
468		15676		5335
469		15676		5335
470		15676		5335
471		15676		5335
472		15676		5335
473		15676		5335
474		15676		5335
475		15676		5335
476		15676		5335
477		15676		5335
478		15676		5335
479		15676		5335
480		15676		5335
481		15676		5335
482		15676		5335
483		15676		5335
484		15676		5335
485		15676		5335
486		15676		5335
487		15676		5335
488		15676		5335
489		15676		5335
490		15676		5335
491		15676	16342	5335
492		15676	16342	5335
493		15676	16342	5335
494		15676	16342	5335
495		15676	16342	5335
496		15676	16342	5335
497		15676	16342	5335
498		15676	16342	5335
499		15676	16342	5335
500	37325	15676	16342	5335
501		15676	16342	5335
502		15676	16342	5335
503		15676	16342	5335
504		15676	16342	5335

505		15676	16342	5335
506		15676	16342	5335
507		15676	16342	5335
508		15676	16342	5335
509		15676	16342	5335
510		15676	16342	5335
511		15676	16342	5335
512		15676	16342	5335
513		15676	16342	5335
514		15676	16342	5335
515		15676	16342	5335
516		15676	16342	5335
517		15676	16342	5335
518		15676	16342	5335
519		15676	16342	5335
520		15676	16342	5335
521		15676	16342	5335
522		15676	16342	5335
523		15676	16342	5335
524		15676	16342	5335
525		15676	16342	5335
526		15676	16342	5335
527		15676	16342	5335
528		15676	16342	5335
529		15676		5335
530		15676		5335
531		15676		5335
532		15676		5335
533		15676		5335
534		15676		5335
535		15676		5335
536		15676		
